# Withdrawn: Parecoxib inhibits inflammatory responses in a mouse model of sepsis

**DOI:** 10.1002/2211-5463.12856

**Published:** 2021-11-29

**Withdrawn**: ‘Parecoxib inhibits inflammatory responses in a mouse model of sepsis’, Yan Sun, Qiaomai Xu, Zhengjie Wu, Yiwen Gong, Lingling Tang. The above article, published online on 3 April 2020 in Wiley Online Library (wileyonlinelibrary.com) as an Accepted Article, has been withdrawn by agreement between the journal Editor‐in‐Chief Miguel De la Rosa, the FEBS Press Editorial Office, and John Wiley and Sons, Ltd. The withdrawal has been agreed because the authors are not responding to requests to finalize their article for publication in the journal as the Version of Record. https://doi.org/10.1002/2211‐5463.12856

